# Effectiveness of using humor appeal in health promotion materials: evidence from an experimental study in Japan

**DOI:** 10.1186/s13690-023-01226-9

**Published:** 2023-12-08

**Authors:** Machi Suka, Takashi Shimazaki

**Affiliations:** Department of Public Health and Environmental Medicine, The Jikei University School of Medicine, 3-25-8 Nishi-Shimbashi, Tokyo, Minato-Ku 105-8461 Japan

**Keywords:** Humor appeal, Persuasiveness, Printable poster, Public health communication

## Abstract

**Background:**

Public health professionals have prepared and distributed many messages and materials to convince the public to adopt healthy behaviors or reduce risky behaviors. However, health promotion materials do not always have the desired effect due to a lack of ability to engage target audience. This study examined the effectiveness of humor appeal (i.e. using humor as an advertising technique to attract attention and increase acceptance of the message) in health promotion materials and how to use it effectively.

**Methods:**

Total 17 printable posters with different frames (loss- vs. gain-framed) × tones (humorous vs. non-humorous) × topics (advance care planning, cancer screening, donor registry, smoking cessation, and physical activity) were created and evaluated for comprehensibility, persuasiveness, and resistance through two web-based surveys. Participants who were Japanese adults aged 25–64 years were randomly assigned one of the posters (200 people each) and asked to rate it. The overall score was calculated as the persuasiveness score (EHPM 2017;22:69) minus the resistance score (EHPM 2022;27:20).

**Results:**

In the advance care planning case, the highest overall score was found in the gain-framed humorous poster, followed by the loss-framed humorous poster, and the non-humorous poster (*p* = 0.007). In the other 4 cases, the posters using humorous illustrations received a significantly lower scores than the non-humorous poster(s).

**Conclusion:**

The use of humor appeal can help improve the acceptability and persuasiveness of the message when dealing with a little-known resistance-prone health topic. Humor appeal will provide an effective hook to direct public attention to what they do not know or care about in public health communication.

**Supplementary Information:**

The online version contains supplementary material available at 10.1186/s13690-023-01226-9.

**Table:** 

Text box 1. Contributions to literature
• Humor appeal is a well-known peripheral cue that can evoke positive feelings in message recipients. Humor appeal may provide an effective hook to direct public attention.
• Research evidence is necessary but not sufficient to judge the pros and cons of humor appeal in public health communication.
• This study suggests that the use of humor appeal can help improve the acceptability and persuasiveness of the message when dealing with a little-known resistance-prone health topic like advance care planning.

## Background

Public health communication has been worked on to encourage people living in the community to take an active role in health promotion and disease prevention [1]. The Centers for Disease Control and Prevention (CDC) published the 10 Essential Public Health Services in 2020 [2]. The third item is ‘Communicate effectively to inform and educate people about health, factors that influence it, and how to improve it’. Public health professionals have prepared and distributed many messages and materials to convince the public to adopt healthy behaviors or reduce risky behaviors.

Unfortunately, health promotion messages and materials do not always have the desired effect. This may be primarily due to a lack of ability to engage target audience. In this age of miscellaneous information, it is becoming increasingly difficult to get the message to the target audience. Moreover, public health communicators need to communicate with everyone regardless of whether or not they are interested in their health. How to direct public attention to what they do not know or care about remains a critical unresolved issue of public health communication.

During the COVID-19 pandemic, accelerating progress in COVID-19 vaccination has been a public health challenge. Wood and Schulman argued for the importance of addressing apathy rather than hesitancy in vaccine promotion messaging [3]. Those with apathy are low-involvement decision-makers, who are more persuaded by quick, catchy, affective, or big picture appeals. This type of information processing termed the peripheral route as opposed to the central route to persuasion in the Elaboration Likelihood Model of Persuasion devised by Petty and Cacioppo [4]. Effective communication strategies for apathetic (low-involvement) people should be very different from those for health-conscious (high-involvement) people. The impact of peripheral cues in a message (e.g. emotional appeals, visual prominence, celebrity endorsement, and source attractiveness) on persuasion cannot be ignored in message design.

Message modality, as well as message design, can be a determinant of effectiveness of public health communication [3, 5]. The Internet has become widely used and is now primary means of communication for the public [6]. However, the Internet is a place where people actively collect and disseminate information of their interest. It is not well suited to deliver information that is unknown or unfamiliar to the public. Accordingly, print media remain heavily used and indispensable for public health communication [7].

We previously reviewed the case of emotional appeal poster made by the Japanese Ministry of Health, Labour, and Welfare and further conducted a comparative study of humorous and non-humorous posters [8, 9]. The results suggested that the application of humorous expression to health promotion materials has the potential to improve their ability to engage target audience. However, humor appeal (i.e. using humor as an advertising technique to attract attention and increase acceptance of the message) has not been very well accepted in the healthcare field [7, 8]. Research evidence is necessary but not sufficient to judge the pros and cons of humor appeal in health promotion materials.

The present study aimed to examine the effectiveness of humor appeal in health promotion materials and how to use it effectively. Total 17 printable posters with different frames (loss- vs. gain-framed) × tones (humorous vs. non-humorous) × topics (advance care planning, cancer screening, donor registry, smoking cessation, and physical activity) were created and evaluated for comprehensibility, persuasiveness, and resistance through two web-based surveys. The results will provide a breakthrough in the development of health promotion materials and also a chance to rethink the conventional wisdom of health communication.

## Methods

### A scoping review

Before starting the study, a scoping review was conducted to have a good understanding of the existing research on humor appeal in health promotion materials. A total of 17 eligible articles were identified from the PubMed database (search query: (humor[Title/Abstract] OR humour[Title/Abstract] OR humorous[Title/Abstract]) AND (messages[Title/Abstract] OR advertisements[Title/Abstract] OR ads[Title/Abstract]) NOT (patients[Title/Abstract]) AND English [Language]) or reference lists of relevant articles. Table 1 summarizes the 17 experimental studies on the effectiveness of humor appeal applied to health promotion materials [10–26]. Most of the studies focused on the effect of humor in video clips targeting young people [14–16, 18–24]. Only 4 studies examined the effect of humor in print materials which aimed to prevent risky behaviors among undergraduate students [10–13]. We found no studies investigating the effectiveness of humor appeal in print materials targeting the general adult population. The present study therefore intended to answer this research question.

**Table 1:** Experimental studies on the effectiveness of humor appeal applied to health promotion materials (a scoping review)

First Author	Journal	Year	Topic	Target	Comparison
【Print ads】					
Iles IA [10]	Health Mark Q	2017	Unprotected sex, drunk driving	Undergraduate	ironic/sarcastic/no humor
Blanc N [11]	Health Commun	2014	Alcohol, tobacco, obesity	Undergraduate	humor/no humor
Soscia I [12]	J Health Commun	2012	Condom	Undergraduate	humor/fear/shock/informative
Lee MJ [13]	J Mass Commun Q	2002	Tobacco	Undergraduate	humor/fear
【Video/TV】					
Ort A [14]	Stud Commun Media	2020	Condom	Undergraduate	humor/fear
Reis ES [15]	Cien Saude Colet	2019	Tobacco	Undergraduate (mostly)	humor/fear
Zhao X [16]	Am J Prev Med	2019	Tobacco	Adolescents	humor/fear
Moyer-Guse E [17]	J Health Commun	2018	Vaccination	Parents of young children	humor/no humor
Lee MJ [18]	Health Promot Pract	2018	Alcohol	Undergraduate	humor/fear
Nabi RL [19]	Health Commun	2016	Self-exams	Undergraduate	humorous/serious
Bleakley A [20]	J Health Commun	2015	Sugary drink	Adolescents	humor/fear/nurturance/control
Lee MJ [21]	J Psychol	2011	Alcohol	Undergraduate	humor/fear
Skalski P [22]	Commun Quarterly	2009	Alcohol	Undergraduate	humor/no humor
Biener N [23]	J Health Commun	2004	Tobacco	Adolescents	humor/illness/control
Conway M [24]	Pers Soc Psychol Bull	2002	Sunscreen, condom	Undergraduate	humor/no humor
【Web/SNS】					
Teoh D [25]	Prev Med Rep	2019	Vaccination	Young adults	humor graphics/infographics/photos
Choi I [26]	JMIR Ment Health	2018	Mental health screening	Adults	humor/no humor

Results for the effectiveness of humor appeal in health promotion materials have been inconsistent across studies. In addition to the differences in target and topic, the type of humor may contribute to the discrepancy. Humor is classified into two major types—playful humor and aggressive humor [27, 28]. Public perception and attitude towards humor often depends on the type of humor. Our previous study suggested that playful humor is more suitable for public health communication because aggressive humor can be controvertial [8]. The present study therefore used playful humor rather than aggressive humor to create the test objects.

### Study outline

We prepared total 17 printable posters with different frames (loss- vs. gain-framed) × tones (humorous vs. non-humorous) × topics (advance care planning, cancer screening, donor registry, smoking cessation, and physical activity) as the test objects. The posters used in this study were shown in Appendix. Each poster consisted of one main message statement and one related illustration, which were placed in accordance with the CDC Clear Communication Index [29].

The main message statements were 22–24 characters long in Japanese as follows. Advance care planning is ‘I want to be myself until the end of my life… So, let’s talk about advance care planning’. Cancer screening is ‘Cancer may creep up on you unawares… So, let’s have cancer screening’. Donor registry is ‘There are people waiting for your help… So, let’s register for the Bone Marrow Donor Program’. Smoking cessation is ‘Tobacco is full of toxins. Let's try to quit smoking together’. Physical activity is ‘Sedentary lifestyle isn't good for your health. Let's move around and refresh your body’. Each message consisted of the consequences (loss) of not taking action in the first half and the benefits (gain) of taking action in the second half. When emphasizing the first half of the message, it was referred to as loss-framed, while when emphasizing the second half of the message, it was referred to as gain-framed [30].

Humor in this study was defined as what provides amusement and provokes laughter. In contrast to prior studies (Table 1), humor was non-verbally expressed by illustrations, because printable posters require the ability to convey a message at a glance. The illustrations were originally painted by one illustrator who fully understood the purposes of the posters. The consequences of not taking action (in loss-framed posters) or the benefits of taking action (in gain-framed posters) were illustrated with humor.

We conducted two web-based surveys among Japanese adults aged 25–64 years in September–November 2022. In the Survey1, total 9 posters for 3 health topics (advance care planning, cancer screening, and donor registry) were evaluated. Loss- and gain-framed humorous posters were compared with a non-humorous poster (as a reference) for each topic. As described later, most people have heard of cancer screening and donor registry at some point, whereas advance care planning is a little-known health topic. The result of Survey1 suggested that humor appeal may not be effective but rather harmful when dealing with a well-known health topic. In order to examine whether this result is consistent and reproducible, we conducted an additional survey. In the Survey2, total 8 posters for 2 common health promotion topics (smoking cessation and physical activity) were evaluated. To make it more detailed, the posters were prepared for a 2 × 2 factorial design (loss-framed humorous, loss-framed non-humorous, gain-framed humorous, and gain-framed non-humorous); target audience were limited to young to middle-aged male office workers; and raters covered both those who were engaged in the risky behavior (i.e. high-involvement) and those who were not (i.e. low-involvement). In Japan, men have a higher smoking rate (27.1% vs. 7.6% in 2019) [31] and a higher mortality of cardiovascular disease (193.8 vs. 110.2, per 100,000 population in 2021) [32] than women. Young to middle-aged men are an important group for lifestyle interventions, [31 and are a suitable audience to receive persuasive messages about smoking cessation and physical activity.

The study protocol was approved by the ethics committees of the Graduate School of Information and Communication (2022–015) and has been conducted in accordance with the Ethical Guidelines for Medical and Biological Research Involving Human Subjects by the Japanese Government.

### Participants

Participants in the surveys were recruited from an online research panel of a leading research company in Japan (Rakuten Insight Inc., Tokyo, Japan). Recruitment emails were sent to randomly selected eligible registrants (Survey1: men and women aged 25–64 years; Survey2: male office workers aged 25–64 years). Participants with serious illness and medical professionals were excluded through a prescreening process. Applicants for participation in the survey were accepted in the order of receipt until the number of participants reached the quotas (Survey1: 100 men and 100 women per poster; Survey2: 100 male office workers with and without the risky behavior, respectively per poster). All participants voluntarily agreed to participate in the survey after reading a description of the purpose and procedure of the survey. Consent to participate was implied by the completion and submission of the survey.

### Measures

Eligible participants were randomly assigned one of the posters. They were asked to see a given poster for at least 15 s and rate it in terms of emotional response, comprehensibility, persuasiveness, resistance, and attention attraction. The online questionnaire forms presented the questions one after one through the operation of a “Next” button. Participants answered one question per page and were unable to go back to the previous page. The components of the questionnaire relevant to this study are detailed below.

#### Emotional response

Participants were asked to what extent the message made them feel 1) surprised, 2) funny 3) fearful, 4) amusing, 5) sad, 6) happy, 7) angry, and 8) unpleasant. Response options were from 1 (not at all) to 5 (extremely) [33, 34]. Because of the lack of established methods of measuring humor perception, humor perception was determined as the average of the amusing and funny scores.

#### Comprehensibility

Participants were asked whether the message was easily understandable. Response options were from 1 (strongly disagree) to 5 (strongly agree). Since humor appeal is expected to encourage the peripheral route to persuasion [4], the primary focus was on ensuring that the recipients find the content easy to understand, rather than determining whether they grasp the content accurately.

#### Persuasiveness and resistance

Participants filled in the persuasiveness and resistance scales which have been proven to be reliable and valid in Japanese people [33, 34]. The persuasiveness score was calculated as the average of 7 items scored on a 1-to-5 point Likert scale; higher scores indicate that the message is more acceptable and persuasive to the audience. The resistance score was calculated as the average of 6 items scored on a 1-to-5 point Likert scale; higher scores indicate that the audience feel greater reactance or apathy to the message. The overall score was calculated as the persuasiveness score minus the resistance score.

#### Attention attraction

Participants were asked ‘When the poster is hanging on a wall at a station yard, what would you do?’ with 4 response options (not become aware/take no notice/throw a glance/stop to look) [34]. The responses were dichotomized into ‘pay attention’ (throw a glance/stop to look) and ‘pay no attention’ (not become aware/take no notice).

In addition to the questions about a given poster, participants were asked whether they were interested in their health (i.e. health awareness) and whether they had been encouraged to do as the poster suggested (i.e. previous contact). They also provided their demographic information (gender, age, marriage, education, occupation, and household income).

### Statistical analysis

All statistical analyses were performed using the SAS ver. 9.4 (SAS Institute, Cary, NC, USA). Significant levels were set at *p* < 0.05. One-way analysis of variance was used to compare the mean scores among the 3 different design posters in the Survey1. Two-way analysis of variance was used to determine the effect of 2 factors (frame and tone) on the scores in the Survey2. The percentages of participants who would pay attention to the poster (i.e. success rate of attention attraction) were compared using Chi-square test.

### Participant and public involvement

The participants in the surveys were not involved in the design, conduct, or reporting of this study.

## Results

### Survey1

Table 2 shows the characteristics of the participants in the Survey1. The percentages of participants who were married (59%) and were employed (71%) were almost equal to that of the Japanese population aged 25–64 years (65% and 73%, respectively in 2020) [35], whereas the percentage of participants with university degrees (47%) was considerably higher than that of the Japanese population (35% in 2020) [35]. Because of random allocation, the 3 groups assigned to the advance care planning, cancer screening, and donor registry posters, respectively had the same distribution of characteristics. About 60% of participants were interested in their health. Very few participants had previous contact with advance care planning compared with cancer screening and donor registry.

**Table 2:** Characteristics of the participants in the Survey1

		Advance care planning		Cancer screening		Donor registry	
Gender	Men: Women	300:300		300:300		300:300	
Age (years old)	25–34	58	10%	57	10%	59	10%
	35–44	114	19%	123	21%	123	21%
	45–54	205	34%	200	33%	211	35%
	55–64	223	37%	220	37%	207	35%
Marriage	Married	357	60%	347	58%	355	59%
	Unmarried	194	32%	192	32%	192	32%
	Divorced/widowed	49	8%	61	10%	53	9%
Education	Compulsory education	10	2%	16	3%	13	2%
	High school	144	24%	172	29%	141	24%
	Junior college/vocational school	157	26%	148	25%	154	26%
	University or higher	289	48%	264	44%	292	49%
Occupation	Full-time job	337	56%	323	54%	339	57%
	Temporary of part-time job	92	15%	94	16%	97	16%
	No occupation	171	29%	183	31%	164	27%
Household income (million yen^a^)	< 2.00	104	17%	93	16%	83	14%
	2.00–5.99	220	37%	257	43%	252	42%
	6.00 +	276	46%	250	42%	265	44%
Health awareness	be interested in own health	378	63%	362	60%	372	62%
Previous contact	have been encouraged to do so	22	4%	134	22%	116	19%

Values are number

^a^1 million yen was about 7,140 U.S. dollars at the time of the survey

Table 3 shows the comparisons of the 3 different design posters in the Survey1. The loss- and gain-framed humorous posters showed significantly higher humor perception scores than the non-humorous poster, indicating that the posters were successfully created to satisfy the prerequisite. In the advance care planning case, the loss- and gain-framed humorous posters were significantly superior to the non-humorous poster in comprehensibility and persuasiveness. Consequently, the overall score changed a negative number (the non-humorous poster) to a positive number (the gain-framed humorous poster) by using humor appeal (Supplementary figure 1). However, in the cancer screening and donor registry cases, the loss- and gain-framed humorous posters were significantly inferior to the non-humorous poster in comprehensibility, persuasiveness, and the overall score. Especially in the donor registry case, the overall score changed a positive number (the non-humorous poster) to a negative number (the gain-framed humorous poster) by using humor appeal (Supplementary figure 1). As show in the scatter plot between the comprehensibility and overall scores of the 9 posters (Supplementary figure 2), the overall score was closely correlated with comprehensibility (Pearson’s correlation coefficient γ = 0.968, *p* < 0.001).

**Table 3:** Comparisons of the 3 different design posters in the Survey1

	Non-humorous	Loss-framed humorous	Gain-framed humorous	*p*
【Advance care planning】				
Humor perception	2.27 (0.87)	2.37 (0.88)	3.03 (1.14)	< 0.001
Comprehensibility	2.63 (1.04)	2.93 (1.04)	3.09 (0.96)	< 0.001
Overall score	-0.31 (1.30)	-0.10 (1.29)	0.10 (1.33)	0.007
Persuasiveness	2.49 (0.88)	2.67 (0.83)	2.79 (0.79)	0.002
Resistance	2.80 (0.82)	2.77 (0.74)	2.69 (0.71)	0.326
【Cancer screening】				
Humor perception	2.13 (0.81)	2.51 (0.94)	2.41 (0.94)	< 0.001
Comprehensibility	3.64 (0.97)	3.62 (0.94)	3.33 (1.05)	0.002
Overall score	0.77 (1.32)	0.63 (1.37)	0.36 (1.43)	0.011
Persuasiveness	3.15 (0.87)	3.03 (0.89)	2.88 (0.92)	0.012
Resistance	2.38 (0.79)	2.40 (0.80)	2.52 (0.85)	0.168
【Donor registry】				
Humor perception	1.83 (0.81)	2.31 (0.84)	2.68 (1.04)	< 0.001
Comprehensibility	3.56 (1.00)	3.07 (1.15)	3.11 (1.10)	< 0.001
Overall score	0.62 (1.20)	0.00 (1.22)	-0.12 (1.29)	< 0.001
Persuasiveness	2.98 (0.77)	2.69 (0.81)	2.53 (0.83)	< 0.001
Resistance	2.36 (0.77)	2.69 (0.78)	2.65 (0.81)	< 0.001

Values are mean (SD)

Humor perception was determined as the average of funny and amusing scores, which were rated on a scale of 1 (not at all) to 5 (extremely)

Comprehensibility was rated on a scale of 1 (strongly disagree) to 5 (strongly agree)

Overall score was the difference between persuasiveness and resistance scores, which were calculated as the average of the scale items scored on a 1-to-5 point Likert scale

The similar tendency was observed in attention attraction. In the advance care planning case, the success rate of attention attraction was significantly higher in the loss-framed humorous poster (51%) and the gain-framed humorous poster (49%) than in the non-humorous poster (39%). However, in the cancer screening and donor registry cases, there were no significant differences among the 3 different design posters; the success rates of attention attraction were 51% in the loss-framed humorous poster, 50% in the gain-framed humorous poster, versus 58% in the non-humorous poster in the cancer screening case (*p* = 0.244), and 55% in the loss-framed humorous poster, 52% in the gain-framed humorous poster, versus 55% in the non-humorous poster in the donor registry case (*p* = 0.785).

### Survey2

Table 4 shows the characteristics of the participants in the Survey2. The percentages of participants who were married and had university degrees were 69% and 70%, respectively. These rates were higher than those of the male Japanese population [35] because the participants were limited to office workers. About 65% of participants were interested in their health. One out of three participants had previous contact with the subjects of the posters, respectively.

**Table 4:** Characteristics of the participants in the Survey2

		Smoking cessation		Physical activity	
Gender	Men: Women	800:0		800:0	
Age (years old)	25–34	38	5%	34	4%
	35–44	98	12%	121	15%
	45–54	319	40%	281	35%
	55–64	345	43%	364	46%
Marriage	Married	549	69%	555	69%
	Unmarried	209	26%	196	25%
	Divorced/widowed	42	5%	49	6%
Education	Compulsory education	3	0%	6	1%
	High school	140	18%	149	19%
	Junior college/vocational school	79	10%	96	12%
	University or higher	578	72%	549	69%
Household income (million yen ^a^)	< 2.00	29	4%	22	3%
	2.00–5.99	244	31%	241	30%
	6.00 +	527	66%	537	67%
Health awareness	be interested in own health	509	64%	524	66%
Previous contact	have been encouraged to do so	273	34%	265	33%

Values are number

^a^1 million yen was about 7,140 U.S. dollars at the time of the survey

Table 5 shows the comparisons of the 4 different design posters in the Survey2. The main effect of tone was significant in humor perception, indicating that the posters were successfully created to satisfy the prerequisite. Both in the smoking cessation and physical activity cases, there was a significant main effect of tone in comprehensibility, persuasiveness, and the overall score; the overall scores were greater than zero but significantly declined by using humor appeal. The main effect of frame and the interaction effect was significant in the overall score as well as persuasiveness in the smoking cessation case.

**Table 5:** Comparisons of the 4 different design posters in the Survey2

	Loss-framed non-humorous	Loss-framed humorous	Gain-framed non-humorous	Gain-framed humorous		p	
					Tone (T)	Frame (F)	F × T
【Smoking cessation】							
Humor perception	2.30 (0.90)	2.53 (0.97)	2.33 (0.99)	2.55 (1.00)	0.001	0.770	0.942
Comprehensibility	3.50 (1.04)	3.49 (1.01)	3.58 (1.01)	3.28 (1.02)	0.027	0.367	0.045
Overall score	0.02 (1.43)	-0.07 (1.25)	-0.01 (1.40)	-0.55 (1.37)	0.001	0.009	0.020
Persuasiveness	2.95 (0.84)	2.85 (0.82)	2.86 (0.87)	2.49 (0.84)	< 0.001	< 0.001	0.020
Resistance	2.93 (0.86)	2.92 (0.71)	2.87 (0.82)	3.03 (0.86)	0.197	0.667	0.143
【Physical activity】							
Humor perception	2.41 (0.93)	2.69 (1.01)	2.62 (0.97)	3.06 (0.98)	< 0.001	< 0.001	0.231
Comprehensibility	3.72 (0.94)	3.37 (1.03)	3.64 (1.01)	3.47 (1.03)	< 0.001	0.859	0.215
Overall score	0.65 (1.33)	0.12 (1.42)	0.67 (1.35)	0.36 (1.50)	< 0.001	0.171	0.262
Persuasiveness	3.14 (0.75)	2.90 (0.90)	3.13 (0.80)	2.92 (0.89)	< 0.001	0.997	0.856
Resistance	2.49 (0.82)	2.79 (0.86)	2.45 (0.80)	2.55 (0.86)	< 0.001	0.022	0.090

Values are mean (SD)

Humor perception was determined as the average of funny and amusing scores, which were rated on a scale of 1 (not at all) to 5 (extremely)

Comprehensibility was rated on a scale of 1 (strongly disagree) to 5 (strongly agree)

Overall score was the difference between persuasiveness and resistance scores, which were calculated as the average of the scale items scored on a 1-to-5 point Likert scale

The success rate of attention attraction tended to be higher in the loss-framed posters. In the smoking cessation case, the highest rate was observed in the loss-framed humorous poster (48%), followed by the loss-framed non-humorous poster (44%), the gain-framed non-humorous poster (38%), and the gain-framed humorous poster (34%) (*p* = 0.025). In the physical activity case, the highest rate was observed in the loss-framed humorous poster (59%), followed by the loss-framed non-humorous poster (55%), the gain-framed humorous poster (51%), and the gain-framed non-humorous poster (43%) (*p* = 0.011).

## Discussion

In order to examine the effectiveness of humor appeal in health promotion materials and how to use it effectively, total 17 printable posters with different frames (loss- vs. gain-framed) × tones (humorous vs. non-humorous) × topics (advance care planning, cancer screening, donor registry, smoking cessation, and physical activity) were created and evaluated for comprehensibility, persuasiveness, and resistance through the two web-based surveys. The overall scores compared among 3–4 different design posters per topic indicated a significant effect of humor appeal to make the message more acceptable and persuasive in the advance care planning case. No such positive effects were observed, but rather the overall scores significantly declined by using humor appeal in the other 4 cases (cancer screening, donor registry, smoking cessation, and physical activity). The effectiveness of humor appeal applied to health promotion materials have been evaluated in various experimental settings (Table 1). Most of the studies focused on a common health promotion topic like alcohol and tobacco and did not discuss the similarities and differences between two or more different types of topics. To our best knowledge, the present study demonstrates for the first time that the effect of humor appeal can differ significantly by message theme.

The following differences can be pointed out between the advance care planning case and the other 4 cases. First, very few participants had previous contact with advance care planning. Accordingly, the non-humorous poster for advance care planning had the lowest score in comprehensibility. Advance care planning may be unknown or unfamiliar to most Japanese people. Second, the non-humorous poster for advance care planning received an overall score of less than zero, meaning that resistance outweighed persuasiveness in the majority of participants. Topics that make them imagine a death scene like advance care planning may be prone to provoke reactance leading to message avoidance and message rejection [36]. Although based on a limited number of experiments, the results of this study suggest that the use of humor appeal can help improve the acceptability and persuasiveness of the message when dealing with a little-known resistance-prone health topic.

Humor appeal has not been very well accepted in the healthcare field. The guidelines for communication program planning from the National Cancer Institute (NCI) recommend “Use a light, humorous approach if appropriate, but pretest to be sure that it works and doesn’t offend the intended audience” [5]. The guidelines for effective writing from the Centers for Medicare and Medicaid Services (CMS) cite humor as a “language to avoid” [37]. It is easy to see how using humor in a serious situation could potentially make the patient uncomfortable. However, public health communication is very different from clinical communication; it is one-to-many communication with people living in the community (versus one-to-one communication between doctor and patient); it mainly addresses health promotion and disease prevention in communities (versus management and care of a patient to combat disease or disorder) [1]. Therefore, the recommendations for clinical communication do not necessarily apply to public health communication.

Public health communicators need to convince everyone to take health promotion and disease prevention actions regardless of whether or not they are interested in their health. These difficulties seem very similar to those in commercial advertisements which aim to make consumers aware of a product or service and convince them to purchase that. According to the Elaboration Likelihood Model of Persuasion devised by Petty and Cacioppo [4], message recipients are more likely to process information in the peripheral route when they have little interest in the subject (i.e. low-involvement). They often make judgments based on peripheral cues in a message, rather than careful and thoughtful consideration of the message itself. Humor appeal is a well-known peripheral cue that can evoke positive feelings in message recipients [38–40]. Humor is known to relax audience, distract audience from counterarguing, and help attract or retain attention to the advertisement [38]. In expectation of these favorable effects, humor appeal has been widely used in commercial advertisements in recent decades [41, 42]. The results of this study suggest that humor appeal can provide an effective hook to direct public attention to the unknown or unfamiliar health topic. The use of humor appeal in public health communication is not to be completely denied. It should be limited to the purpose of directing public attention to what they do not know or care about.

Compared with advance care planning, cancer screening, donor registry, smoking cessation, and physical activity have been well covered in advertisements. When people have heard and seen the same kinds of messages many times, they are likely to feel fatigued towards the messages [43]. It was initially expected that the use of humor appeal would refresh the well-known health messages to prevent message fatigue to some extent, but the results were contrary to our expectations. The decline in the overall score by using humor appeal in the 4 cases could be due to decreasing comprehensibility. Participants may have got confused or distracted if the illustrations were not perceived as related to the message. Visuals in a public communication material should be clearly designed to help convey the message easily and quickly [29]. This is especially important for printable posters in which a message must be expressed in limited words and visuals.

The comparison of 3 posters for advance care planning in the Survey1 showed that the overall score was the highest in the gain-framed humorous poster, followed by the loss-framed humorous poster. The comparisons of 4 posters for smoking cessation and physical activity, respectively in the Survey2 were a 2 × 2 factorial design to determine the effect of 2 factors (frame and tone) on the overall scores. A significant interaction effect was observed in the smoking cessation case, but not in the physical activity case. These contradictory results alone do not allow us to conclude whether the effect of humor appeal can be influenced by messaging frame. To our best knowledge, no prior studies have compared the effectiveness of humor appeal in different frames. Further studies are needed to clarify the interaction between humor appeal and messaging frame and provide suggestions on how to use humor appeal effectively in health promotion materials.

The results of this study can provide a breakthrough in the development of health promotion materials and also a chance to rethink the conventional wisdom of health communication. On the contrary, this study has the following potential limitations. First, this study compared 3–4 different design posters per topic. Only one poster was created for each frame × tone pattern for each topic. Although the findings from the Survey1 were consistently confirmed by the Survey2, additional experimental studies of various health promotion materials are needed to ensure the generalizability of findings. Second, humorous illustrations are multi-colored and three-dimensional, while non-humorous illustrations are one-colored and two-dimensional. Our previous study suggested that formatted messages are more likely to be perceived as attractive and helpful by the audience and more likely to increase the willingness to read than unformatted messages [44]. The effect of using humorous illustrations may have been somewhat influenced by color, shape, and other visual design elements. Third, humor perception was determined by the emotional responses of ‘amusing’ and ‘funny’. Humor is universally used to provide amusement and provoke laughter despite cultural differences in humor perception [45]. The combination of amusing and funny ratings seems to have captured the core of the humor, but may not have captured the humor as a whole. Fourth, the participants in the surveys were recruited from an online research panel. People who cannot access the website through computers or smartphones had no opportunity to participate in the surveys. The results of this study may not have reflected responses from a lower socioeconomic group.

## Conclusion

Many health promotion materials have been developed and distributed to the public to convince them to adopt healthy behaviors or reduce risky behaviors. Humor appeal has been widely used in commercial advertisements in recent decades because humor has a potential to attract attention and increase acceptance of the message. This study compared printable posters with different frames (loss- vs. gain-framed) × tones (humorous vs. non-humorous) × topics (advance care planning, cancer screening, donor registry, smoking cessation, and physical activity). The posters using humorous illustrations received a significantly higher scores than the non-humorous poster in the advance care planning case, but did not in the cancer screening, donor registry, smoking cessation, and physical activity cases. The results of this study suggest that the use of humor appeal can help improve the acceptability and persuasiveness of the message when dealing with a little-known resistance-prone health topic. Humor appeal will provide an effective hook to direct public attention to what they do not know or care about in public health communication.

### Supplementary Information

**Additional file 1:** **Appendix.** Posters used in the study.

**Additional file 2: Supplementary figure 1.** Mean overall scores of the 9 posters for 3 topics in the Survey 1.

**Additional file 3: Supplementary figure 2. **Scatter plot with a linear trend line between the comprehensibility and overall scores of the 9 posters in the Survey1.

## Data Availability

The dataset of this study will not be shared because the Ethical Guidelines prohibit researchers from providing their research data to other third-party individuals.

## Abbreviations

CDC: Centers for Disease Control and Prevention
CMS: Centers for Medicare and Medicaid Services
NCI: National Cancer Institute
