# Supplementary material for: Effectiveness of using humor appeal in health promotion materials: evidence from an experimental study in Japan
Source: Arch Public Health. 2023 Dec 8;81:212. doi: 10.1186/s13690-023-01226-9 (PMC10704777; doi:10.1186/s13690-023-01226-9)
Supplement: Supplementary file 1 — Additional file 1: Appendix. Posters used in the study. [file 13690_2023_1226_MOESM1_ESM.pdf]

【Advance care planning】

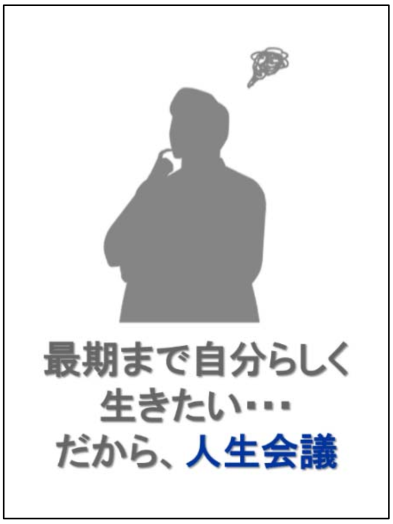

Non-humorous

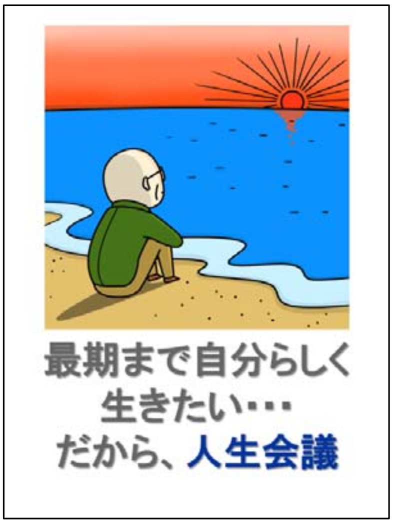

Loss-framed humorous

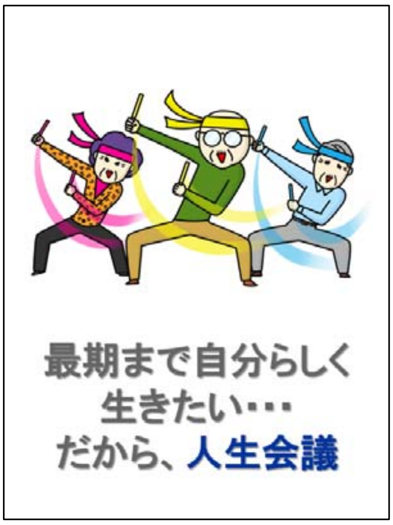

Gain-framed humorous

【Cancer screening】

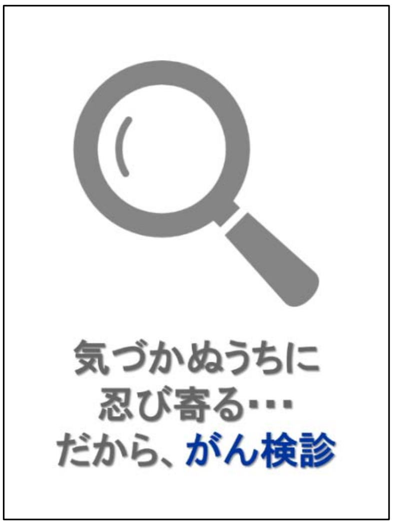

Non-humorous

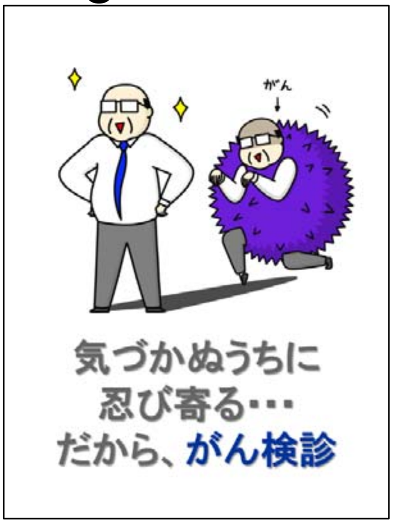

Loss-framed humorous

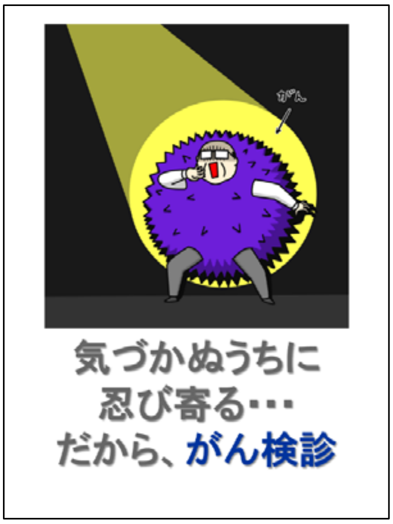

Gain-framed humorous

【Donor registry】

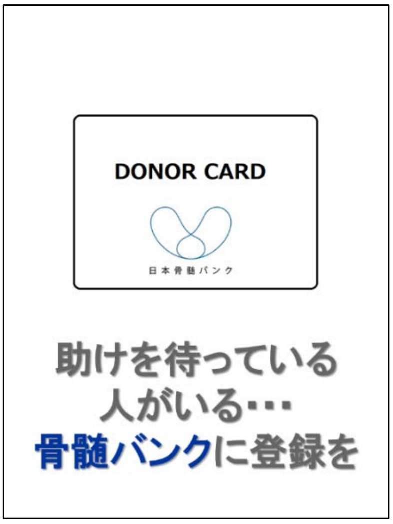

Non-humorous

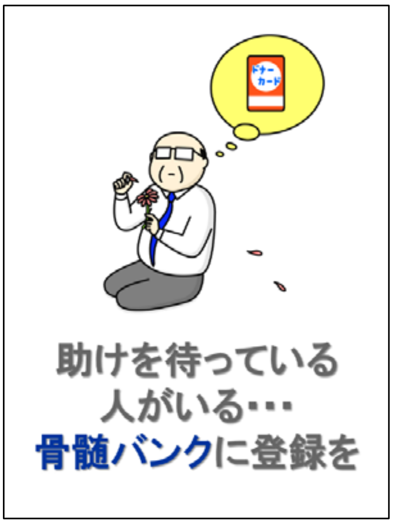

Loss-framed humorous

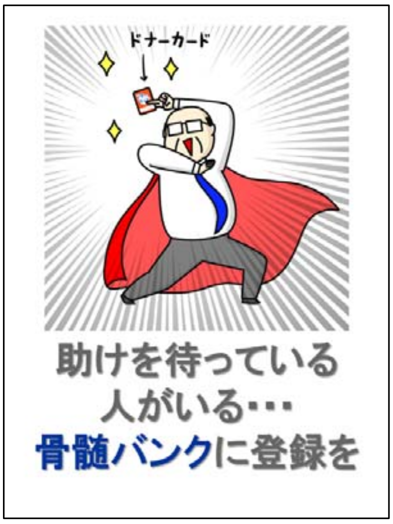

Gain-framed humorous

#【Smoking cessation】

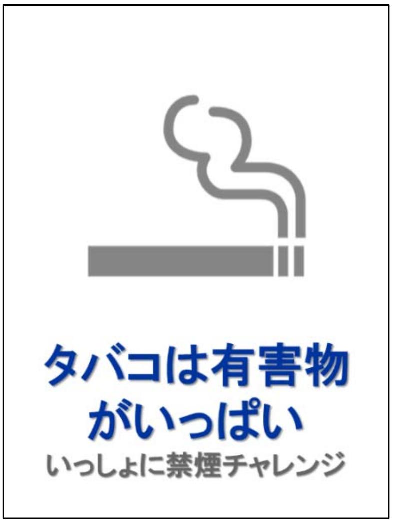

Loss-framed non-humorous

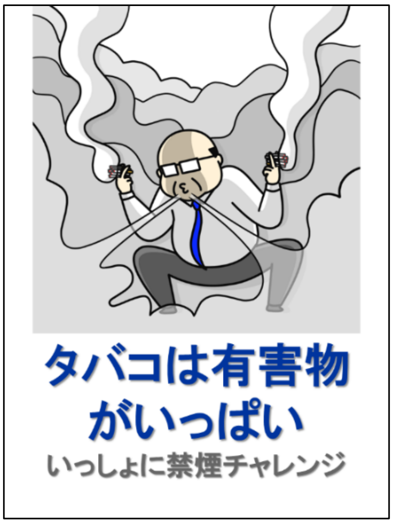

Loss-framed humorous

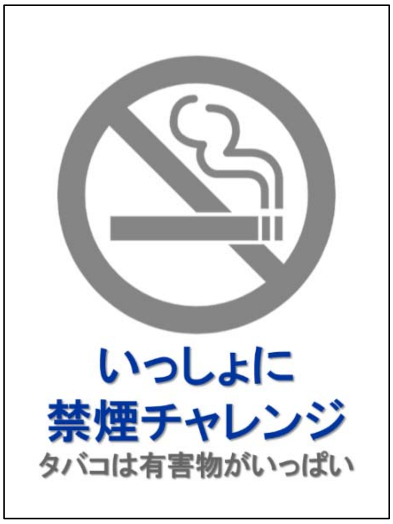

Gain-framed non-humorous

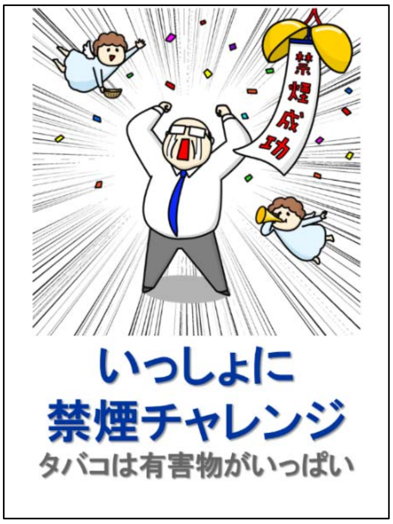

Gain-framed humorous

#【Physical activity】

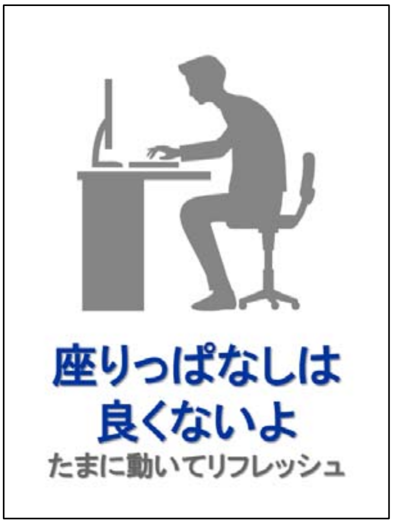

Loss-framed non-humorous

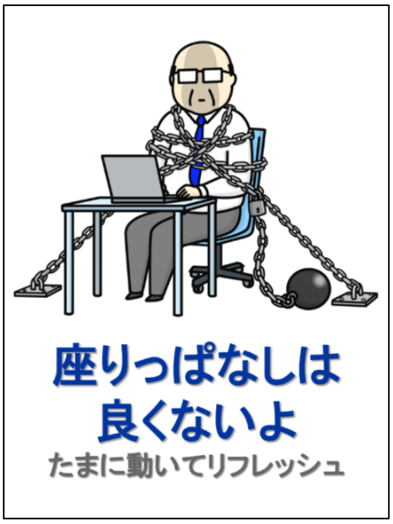

Loss-framed humorous

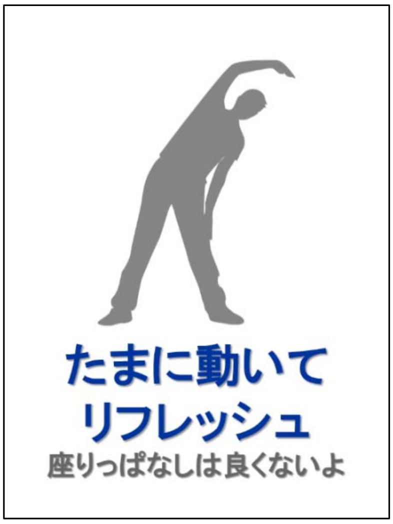

Gain-framed non-humorous

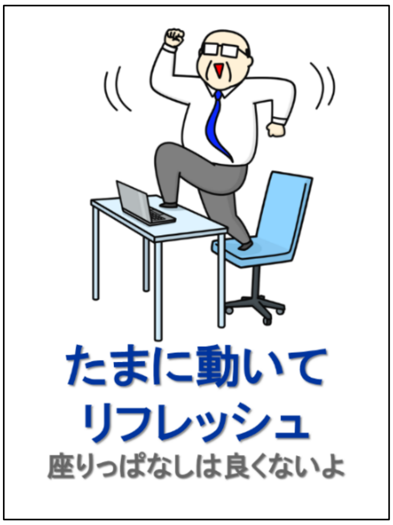

Gain-framed humorous
