# Supplementary figures and images for: Effectiveness of using humor appeal in health promotion materials: evidence from an experimental study in Japan
Source: Arch Public Health. 2023 Dec 8;81:212. doi: 10.1186/s13690-023-01226-9 (PMC10704777; doi:10.1186/s13690-023-01226-9)

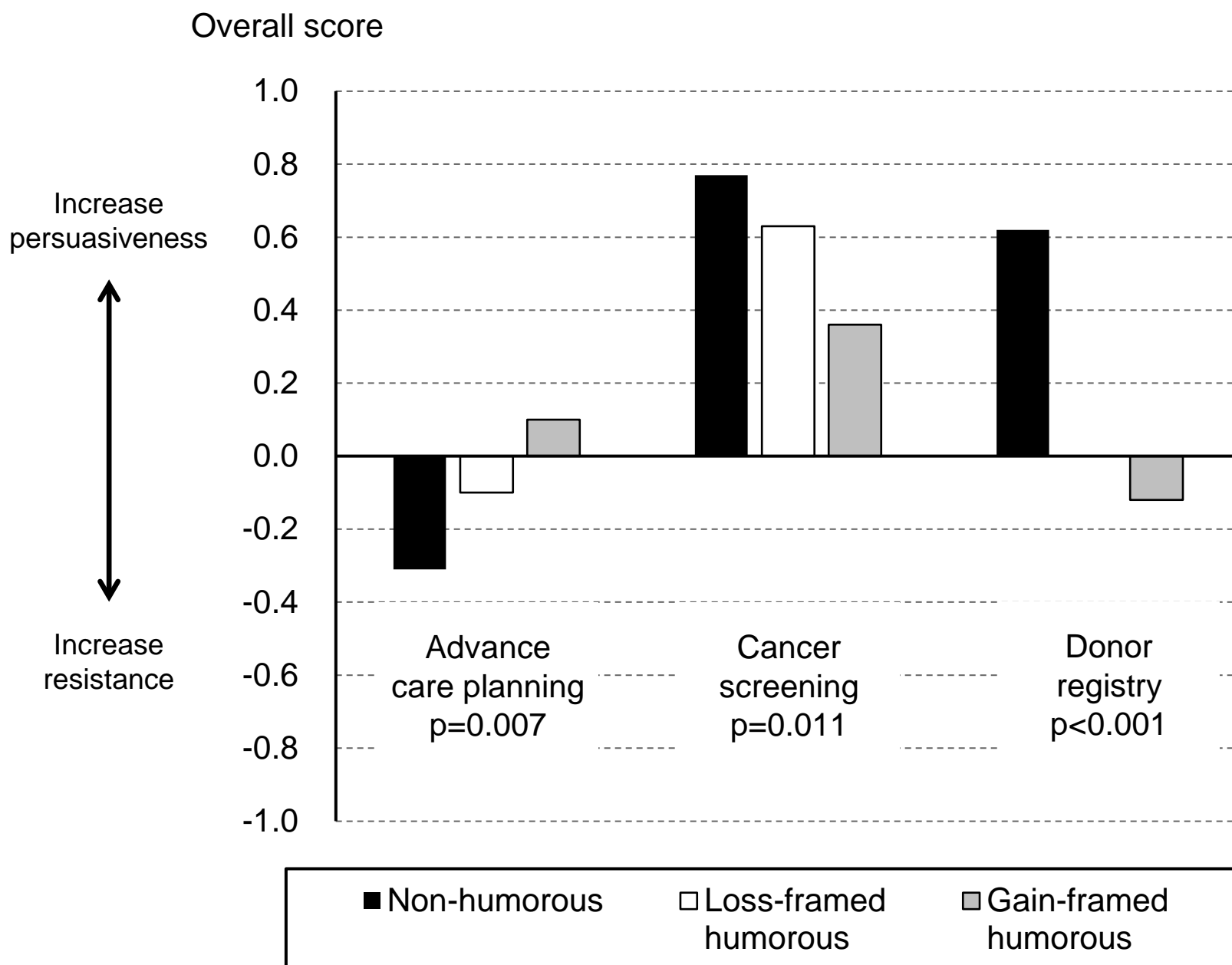

Supplementary figure1.

Mean overall scores of the 9 posters for 3 topics in the Survey1.

Supplement: Supplementary file 2 — Additional file 2: Supplementary figure 1. Mean overall scores of the 9 posters for 3 topics in the Survey 1. [file 13690_2023_1226_MOESM2_ESM.pdf]
