# Supplementary material for: Effectiveness of using humor appeal in health promotion materials: evidence from an experimental study in Japan
Source: Arch Public Health. 2023 Dec 8;81:212. doi: 10.1186/s13690-023-01226-9 (PMC10704777; doi:10.1186/s13690-023-01226-9)
Supplement: Supplementary file 3 — Additional file 3: Supplementary figure 2. Scatter plot with a linear trend line between the comprehensibility and overall scores of the 9 posters in the Survey1. [file 13690_2023_1226_MOESM3_ESM.pdf]

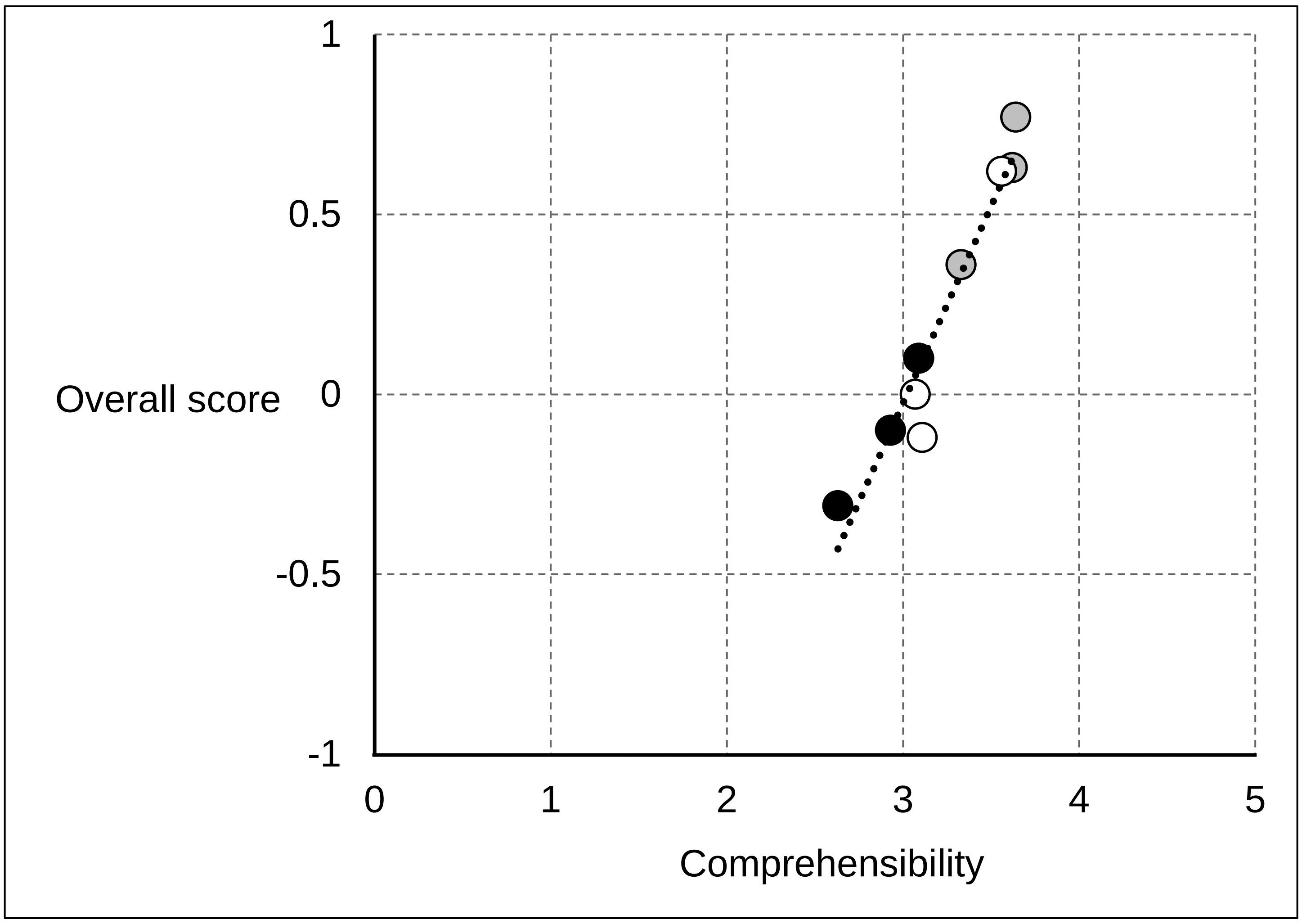

Supplementary figure2.
Scatter plot with a linear trend line between the comprehensibility and overall scores of the 9 posters in the Survey1.
Dots represent the mean comprehensibility and overall scores of the poster: black=advance care planning, gray=Cancer screening, white=Donor registry.
Pearson’s correlation coefficient γ=0.968 ($p<0.001$)
